# Comparison of Surgical Times for Gonio Lenses and Viewing Systems in iStent Inject^®^ W Surgery

**DOI:** 10.3390/jcm14113680

**Published:** 2025-05-24

**Authors:** Tatsuya Fujii, Yoshihito Sakanishi, Shuu Morita, Nobuyuki Ebihara

**Affiliations:** Department of Ophthalmology, Juntendo University Urayasu Hospital, Ciba 279-0021, Japan; ysakani@juntendo.ac.jp (Y.S.); s-morita@juntendo.ac.jp (S.M.); ebihara@juntendo.ac.jp (N.E.)

**Keywords:** surgical time comparison, iStent implantation, gonio lens systems

## Abstract

**Background/Objectives**: The aim of the study was to investigate the difference in operative times for iStent inject^®^ W (iStent) implantation based on variations in the gonio lens and with use of a surgical microscope or the NGENUITY^®^ 1.5 (Alcon Laboratories, Inc. Fort Worth, TX, USA) which is a digitally assisted surgery system). **Methods**: This retrospective study included patients who underwent iStent implantation performed by the same surgeon at Juntendo University Urayasu Hospital between January 2021 and December 2022. Participants were divided into Group M, which utilized a disposable gonio lens (Ocular Instruments, Washington, DC, USA) and the surgical microscope; Group A, which used Ahmed’s DVX Surgical Gonio lens^®^ (Ocular Instruments) and the surgical microscope; and Group D, which employed the disposable gonio lens and the NGENUITY^®^ system. The preparation and recovery times were retrospectively compared as the primary outcome across the three groups. **Results**: Preparation and recovery times were significantly shorter in Group A than those in the other two groups (both *p* < 0.01). There were no significant differences in preparation (*p* = 0.968) and recovery times (*p* = 0.841) between groups M and D. **Conclusions**: In iStent surgery, preparation and recovery times for angle observation were shorter with the Ahmed gonio lens than with the disposable gonio lens. No difference was observed between the surgical microscope and digitally assisted surgery methods. The Ahmed gonio lens is useful for surgeons who perform cataract surgery through an upper incision.

## 1. Introduction

Glaucoma is the leading cause of blindness in Japan. The Tajimi Study reported a 3.9% prevalence rate of primary open-angle glaucoma [1]. Although it is standard practice to initially reduce intraocular pressure (IOP) using glaucoma eye drop therapy, when the eye drops are insufficient to produce this effect, surgical intervention is considered. Recently, compared to trabeculectomy, minimally invasive glaucoma surgery (MIGS) has become more widely used because of its scar-sparing approach. The iStent^®^ (Glaukos Corporation, Laguna Hills, CA, USA) was one of the first MIGS devices, introduced by Spiegel et al. in 2007 [2]. In 2012, it was approved by the U.S. Food and Drug Administration for use in surgery simultaneously with cataract surgery in patients with early to intermediate-stage open-angle glaucoma treated with eye drops. In Japan, the guidelines for using intraocular drainage with cataract surgery were established in 2016, with a second edition released in 2019, and the criteria for iStent^®^ use were significantly expanded in the second edition [3]. Following this expansion, iStent^®^ is now used during cataract surgery in cases of early- and intermediate-stage glaucoma and cataract progression.

**Figure 1:**
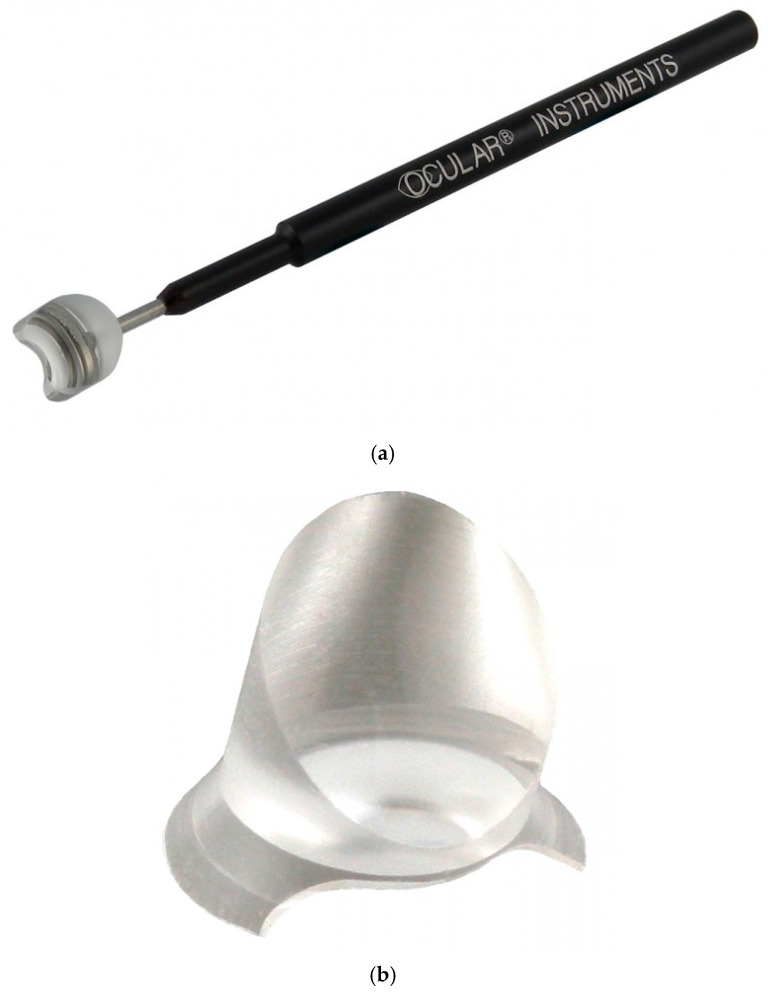

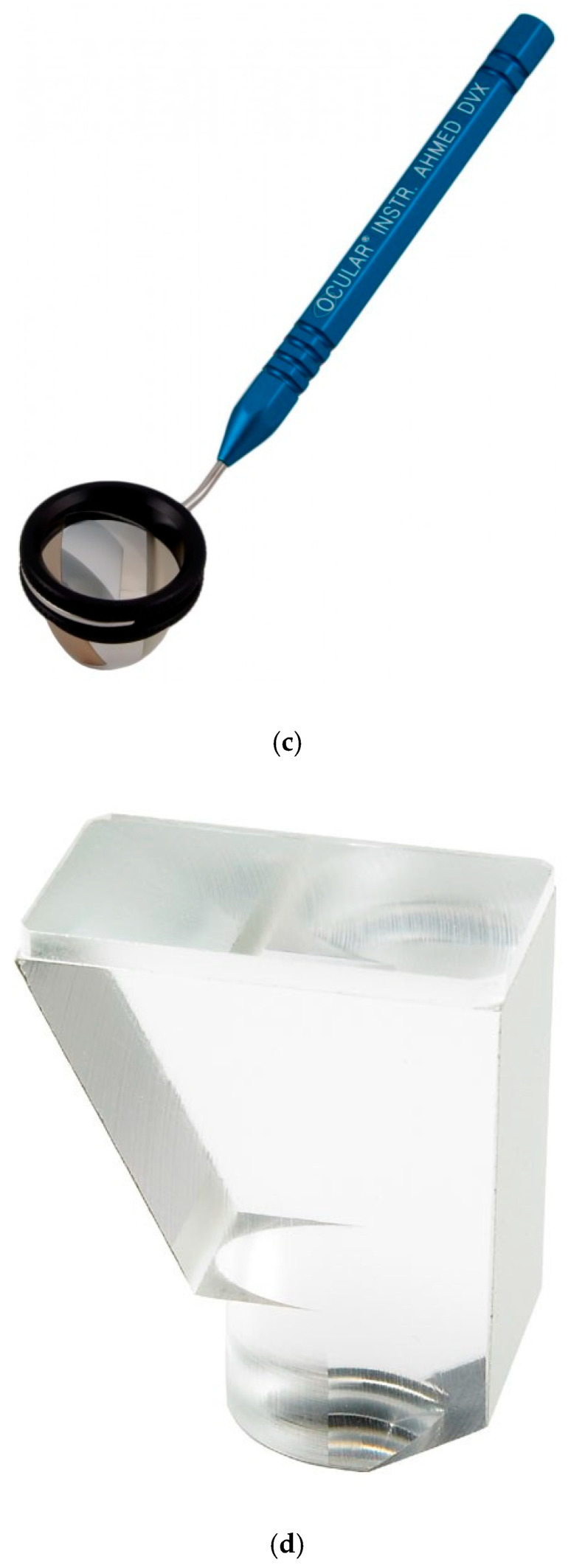
Typical surgical gonio lenses. (**a**) Swan–Jacob lens; (**b**) disposable hands-free surgical gonio lens; (**c**) Ahmed DVX surgical gonio lens; (**d**) Mori goniotomy lens.

Implantation of the iStent^®^ requires placing a gonio lens on the cornea for angle observation, with an inclination of approximately 70° to the visual axis. For direct gonioscopy that requires the surgeon to tilt the microscope or the patient’s head, the Swan–Jacob gonio lens (Ocular Instruments, Washington, DA, USA) or the disposable hands-free surgical gonio lens (Ocular Instruments, Washington, DA, USA) (hereafter referred to as the “disposable gonio lens”) can be used. Double-mirror gonio lenses that allow observation of the angle without tilting the microscope include the Mori gonio lens (Ocular Instruments, Washington, DA, USA) (hereafter referred to as “Mori gonio lens”) and Ahmed’s DVX Surgical Gonio lens^®^ (Ocular Instruments, Washington, DA, USA) (hereafter referred to as “Ahmed gonio lens”) (reprinted from R.E. Medical Corporation, “Surgical Lens Catalog”, which compares typical gonio lenses, as shown in Figure 1 and Table 1). The Mori goniotomy lens has a magnification of 0.8× and a 110° field of view, enabling angled observation with a direct view under the surgical microscope [4]. Ahmed gonio lens^®^ has a magnification of 1.3× and a 120° field of view, superior to those of the disposable and the Mori gonio lens, enabling angle observation under a microscope with a frontal view. Few reports exist on the use of the Ahmed gonio lens. The only study on its use for dissecting angle adhesions in patients with primary angle-closure glaucoma was published by Lei et al. in 2022 [5].

Digital surgery, also known as “heads-up surgery” or “digitally assisted surgery (DAS)”, is performed by viewing the surgical field projected onto a 3D monitor. In DAS, the surgeon does not need to look through the surgical microscope; therefore, the microscope and the surgeon’s line of sight do not need to align. DAS can also be used in NGENUITY^®^ digital surgery to process images of the surgical field, enhancing the observation of the angle during the MIGS procedure, as with the iStent. Despite the increasing use of MIGS, there is limited information on the impact of varying gonio lenses and observation systems on the operating time and outcomes.

Therefore, we aimed to address this gap by investigating the difference in operative times for iStent inject^®^ W (hereafter referred to as “iStent”) implantation with varying gonio lenses and viewing systems.

## 2. Materials and Methods

### 2.1. Case Background

This study was approved by the Ethics Committee of Juntendo University Urayasu Hospital and conducted following the principles of the Declaration of Helsinki. Informed consent was obtained from all patients.

In this retrospective study, we included cases of iStent implantation performed by the same surgeon (Y.S.) at Juntendo University Urayasu Hospital between January 2021 and December 2022. The cases were divided into three groups: Group M, which used the disposable gonio lens and microscope oculars; Group A, which used the Ahmed gonio lens and lens barrel; and Group D, the DAS group, which used the disposable gonio lens and NGENUITY^®^ 1.5(Alcon Laboratories, Inc. Fort Worth, TX, USA). The study included 24 eyes from 24 patients in Group M (mean age: 74.4 ± 6.4 years), 25 eyes from 25 patients in Group D (mean age: 71.4 ± 10.7 years), and 19 eyes from 19 patients in Group A (mean age: 73.0 ± 6.6 years). Preparation and recovery times were evaluated from the surgical videos and compared retrospectively.

We reprinted the “Surgical Lens Catalog” from R.E. Medical Corporation, comparing typical gonio lenses in Figure 1 and Table 1.

### 2.2. Surgical Method

A 1.00 mm side port was created in all three groups, the anterior chamber was formed using the DisCoVisc 1.0^®^ (Nihon Alcon K.K. Tokyo, Japan) viscoelastic agent, and a 2.40 mm main upper incision was made. The anterior capsule was then incised, and phacoemulsification and cortical aspiration were performed, followed by intraocular lens implantation. After injecting the viscoelastic substance and forming the anterior chamber, the iStent was implanted in each group: in Group M, after injecting the viscoelastic substance into the anterior chamber, the surgeon moved to the patient’s ear side, adjusted the microscope barrel tilt, changed the patient’s head position, and repositioned the microscope in the XYZ direction. The disposable gonio lens was then placed to visualize the angle, the iStent was inserted with both hands through the side port on the ear side, and the anterior chamber was washed. In Group A, after injecting the viscoelastic material into the anterior chamber, the Ahmed gonio lens was used to visualize the angle, and the iStent was inserted with one hand, followed by anterior chamber irrigation. In Group D, after injecting the viscoelastic substance into the anterior chamber, the microscope oculars were rotated toward the ear, the microscope oculars’ tilt was adjusted, and the patient’s head position and microscope alignment in the XYZ direction were modified. The disposable gonio lens was placed to visualize the angle, the iStent was inserted with both hands through the side port on the ear side, and the anterior chamber was washed.

The preparation time was defined as the interval from the end of cataract surgery (when the viscoelastic injection needle was removed from the eye) to the moment the anterior chamber angle could be visualized under the microscope. The recovery time was defined as the interval from completing iStent implantation (when the injector was removed from the anterior chamber) to when the anterior chamber was visible under the microscope. Preparation and recovery times were the primary outcomes, and other metrics (e.g., safety, complications) were not evaluated in this study. The procedures for preparation and recovery in each group are presented in Table 2 and Table 3.

### 2.3. Statistics

Continuous variables in the case backgrounds were analyzed using the Kruskal–Wallis test, while binary nominal variables were analyzed using Fisher’s exact test. The Bonferroni correction was used when comparing preparation and recovery times. Statistical significance was set at *p* < 0.05, and the analysis was performed using IBM SPSS (v26 [for Mac]) (IBM Corp., Armonk, NY, USA).

## 3. Results

A total of 68 patients (24 in Group M, 19 in Group A, and 25 in Group D) were included in the study. The demographic characteristics of each group are presented in Table 4. The mean age of the patients was 74.4 ± 6.4 years in Group M, 73.0 ± 6.6 years in Group A, and 71.4 ± 10.7 years in Group D, with no significant difference between the groups. There was no significant difference in the sex distribution among the study groups. There was no significant difference in the number of right and left eyes among all groups.

The preparation and recovery times for each group are shown in Table 5. The preparation time was 54.5 ± 18.2 s in Group M, 20.9 ± 5.6 s in Group A, and 51.6 ± 14.1 s in Group D. The recovery time was 19.5 ± 5.9 s in Group M, 9.6 ± 4.6 s in Group A, and 20.0 ± 5.3 s in Group D. In all cases, Group A had significantly shorter preparation (*p* < 0.01) and recovery (*p* < 0.01) times than groups M and D. There were no significant differences between groups M and D in the preparation (*p* = 0.968) and recovery (*p* = 0.841) times.

## 4. Discussion

In this study, we examined the difference in preparation and recovery times between iStent implantation using a gonio lens and viewing system. We found that preparation and recovery times were shorter with the Ahmed gonio lens than with the disposable gonio lens and DAS. In iStent implantation, using a disposable gonio lens requires the surgeon to move to the patient’s ear side and reposition the patient’s head. In contrast, with the Ahmed gonio lens, these steps are not needed, allowing for a shorter procedure time. There was no significant difference in preparation and recovery times between when NGENUITY^®^ was used with a disposable gonio lens and when a surgical microscope was used with a gonio lens. However, the preparation and recovery times were significantly shorter in the group using the Ahmed gonio lens.

Double-mirror-type gonio lenses, such as the Ahmed gonio lens, have reduced resolution and a narrower field of view owing to double image reflection. However, the Ahmed gonio lens surpasses the Mori gonio lens [3] in magnification and field of view. Clear angle observation is essential for iStent surgery and other angle-related MIGS procedures [6]. This study suggests that the Ahmed gonio lens may reduce iStent implantation times, even for surgeons performing phacoemulsification through an upper incision without switching to a lateral position. Similar to the Mori gonio lens’ usefulness for trabeculectomy and other angle surgeries [4], the Ahmed gonio lens is considered beneficial for iStent^®^ surgery and other angle procedures.

DAS performed using NGENUITY^®^ has been reported in vitreous surgery, cataract surgery, and trabeculectomy [7,8,9]. The advantages of digital surgery with NGENUITY^®^ include natural image reproduction with a high dynamic range, a wide depth of field, lower intraocular illumination levels than conventional microscopes [10], and enhanced visibility through image processing of the surgical field. In MIGS procedures for the trabecular meshwork, such as iStent, improved visualization enhances the contrast between the trabecular meshwork and the surrounding tissue. With a disposable gonio lens, the surgeon must move to the ear side; however, with NGENUITY^®^, the surgeon can insert the iStent by simply rotating the telescope while remaining in the upper position. In this study, there was no significant difference in preparation or recovery times between when NGENUITY^®^ was used with a disposable gonio lens and when a surgical microscope was used with a gonio lens. However, in canal-based MIGS, NGENUITY^®^ offers an advantage in terms of angle visualization and digital image enhancement [11].

When using the Ahmed gonio lens for angle observation, the gonio lens must be held in the left hand. The iStent injector is held in the right hand for the right eye and in the left hand for the left eye. Training is essential to master the operation. In 2003, Ayaki et al. studied the learning curve for performing phacoemulsification and aspiration with a lateral incision in five ophthalmologists. They examined the learning curve for making a lateral corneal incision, performing phacoemulsification and aspiration, and inserting the intraocular lens, using the right hand for the right eye and the left hand for the left eye [12]. For first-time left-hand surgery, experience with other surgical techniques using the right hand seemed difficult to apply. This trend in the learning curve for the left hand seemed to align with the progress of beginners. The iStent inject is designed to enhance procedural efficiency and ease the learning curve for successful implantation [13]. However, if a surgeon struggles to perform iStent implantation stably with one hand, using a disposable gonio lens that allows angle observation without needing the other hand may be an option. Over time, one-handed implantation could become feasible as the surgeon gains proficiency.

This study has some limitations. We only examined surgeries involving an upper incision and did not assess the impact of different angle lenses on operating time using a lateral incision. Additionally, the time required for iStent implantation was not considered, and further investigation into this aspect will be necessary. Moreover, this was a retrospective study with a small sample size. In addition, the surgeon’s proficiency with the gonio lens and viewing system may have affected the results.

## 5. Conclusions

Using the Ahmed gonio lens in iStent implantation reduced the preparation and recovery times for angle observation when compared with those using disposable gonio lenses. The Ahmed gonio lens is useful for surgeons who perform cataract surgery using an upper incision.

## Figures and Tables

**Table 1:** Comparison of typical surgical gonio lenses.

	Swan–Jacob	Disposable	Ahmed DVX	Mori
Microscope tilt	70°	70°	Unnecessary	Unnecessary
Magnification	1.2×	1.2×	1.3×	0.8×
View angle (°)	90	90	120	110
Eyepiece (mm)	9.5	15.5	10.0	11.5
Handle	With	Without	With	Without
Cost ^※1^	USD 650.00	USD 30.00/unit ^※2^	USD 1050.00	USD 850.00

^※1^ The cost of each gonio lens from R.E. Medical Corporation was accurate at May 16 2025. ^※2^ The disposable gonio lens is sold in a carton of 10.

**Table 2:** Procedures for preparing each group.

	Group M	Group A	Group D
Removal of viscoelastic material injection needle from inside the eye	〇	〇	〇
The surgeon moves to the temporal side	〇		
Tilt the microscope	〇		〇
Change in patient’s head position	〇		〇
Adjusting the position of the microscope	〇		〇
Put the gonio lens on the cornea	〇	〇	〇
Observe the angle	〇	〇	〇

〇 The procedure is necessary.

**Table 3:** Procedures for recovery in each group.

	Group M	Group A	Group D
Removal of iStent inject W^®^ from the anterior chamber	〇	〇	〇
The surgeon moves upward	〇		
Change in patient’s head position	〇		〇
Restore the tilt of the microscope	〇		〇
Adjusting the position of the microscope	〇		〇
Observe the anterior chamber	〇	〇	〇

〇 The procedure is necessary.

**Table 4:** Patient background.

	Group M	Group A	Group D	*p*-Value
No. (eye)	24	19	25	
Age (years)	74.4 ± 6.4	73.0 ± 6.6	71.4 ± 10.7	0.62
Male: Female	11:13	8:11	17:8	0.17
Right: Left (eyes)	13:11	9:10	12:13	0.91

Kruskal–Wallis test with Bonferroni correction.

**Table 5:** Preparation and recovery times for each group.

	Group M	Group A	Group D	*p*-Value
Preparation time (seconds)	54.5 ± 18.2	20.9 ± 5.6	51.6 ± 14.1	<0.01
Recovery time (seconds)	19.5 ± 5.9	9.6 ± 4.6	20.0 ± 5.3	<0.01

Kruskal–Wallis test with Bonferroni correction.

## Data Availability

The raw data supporting the conclusions of this article will be made available by the authors on request.

## Abbreviations

The following abbreviations are used in this manuscript:

SPSS	Statistical Package for the Social Sciences
PEA	Phacoemulsification and Aspiration
IOP	Intraocular Pressure
MIGS	Minimally Invasive Glaucoma Surgery
DAS	Digitally Assisted Surgery
